# The pathogenesis, diagnosis, prevention, and treatment of CAR-T cell therapy-related adverse reactions

**DOI:** 10.3389/fphar.2022.950923

**Published:** 2022-10-14

**Authors:** Yanping Li, Yue Ming, Ruoqiu Fu, Chen Li, Yuanlin Wu, Tingting Jiang, Ziwei Li, Rui Ni, Li Li, Hui Su, Yao Liu

**Affiliations:** Department of Pharmacy, Daping Hospital, Army Medical University, Chongqing, China

**Keywords:** CAR-T cell therapy, cytokine release syndrome, ICANS, consensus grading, organ system toxicity, treatment strategies

## Abstract

Chimeric antigen receptor (CAR)-T cell therapy is effective in the treatment of refractory/relapsed (r/r) hematological malignancies (r/r B-cell lymphoblastic leukemia, B-cell lymphoma, and multiple myeloma). In addition, it is being explored as a treatment option for solid tumors. As of 31 March 2022, seven CAR-T therapies for hematological malignancies have been approved worldwide. Although CAR-T therapy is an effective treatment for many malignancies, it also causes adverse effects. The incidence of cytokine release syndrome (CRS), the most common adverse reaction after infusion of CAR-T cells, is as high as 93%.CRS, is the leading risk factor of immune effector cell-associated neurotoxicity syndrome (ICANS), as well as cardiovascular, hematological, hepatorenal, skin, pulmonary, and gastrointestinal toxicity. Severe adverse reactions complicated by CRS severely impede the widespread application of CAR-T therapy. The CAR-T product was initially approved in 2017; however, only limited studies have investigated the adverse reactions owing to CAR-T therapy compared to that of clinically approved drugs. Thus, we aimed to elucidate the mechanisms, risk factors, diagnostic criteria, and treatment of toxicities concurrent with CRS, thereby providing a valuable reference for the safe, effective, and widespread application of CAR-T therapy.

## Introduction

Chimeric antigen receptor (CAR)-T cell therapy has gained attention as an effective treatment for related tumors owing to the unsatisfactory efficacy of conventional chemoimmunotherapy and radiotherapy for most relapsed/refractory (r/r) hematological malignancies. Seven CAR-T therapies approved including tisagenlecleucel, axicabtagene ciloleucel, lisocabtagene maraleucel, brexucabtagene autoleucel and relmacabtagene autoleucel (these five target CD-19), idecabtagene vicleucel and ciltacabtagene autoleucel (these two target B cell maturation antigen [BCMA]), have been approved globally (Table 1) (Neelapu et al., 2017; Maude et al., 2018; Schuster et al., 2019; Abramson et al., 2020; Berdeja et al., 2021; Munshi et al., 2021; Shah et al., 2021; Westin et al., 2021; Ying et al., 2021). Currently, relmacabtagene autoleucel has been approved in China only. CAR-T therapies have currently been approved for the treatment of hematological malignancies including r/r B-lymphoblastic leukemia (r/r B-ALL), r/r B-cell non-Hodgkin lymphoma (NHL), and r/r multiple myeloma (r/r MM) (Maude et al., 2018; Abbasi et al., 2020; Abramson et al., 2020; Berdeja et al., 2021; Jain et al., 2021; Munshi et al., 2021; Ying et al., 2021).

**TABLE 1:** Approved CAR-T cell therapy.

Name (trade name)	Company	Target antigen	CAR construct (Crees and Ghobadi, 2021; Anderson, 2022)	Listing date	Indication	
Tisagenlecleucel (Kymriah)	Novartis	CD19	Second generation, CD3ζ+4-1BB Lentiviral vector	FDA 2017.08.30 EMA 2018.08.27	Paediatric and young adult patients (age 3–25 years) with r/r B-ALL; adult (≥18 years) patients with r/r DLBCL (Braendstrup et al., 2020)	
				FDA 2022.05.27	Adult patients with r/r FL (Fowler et al., 2022)	
Axicabtagene ciloleucel (Yescarta)	Kite pharma	CD19	Second generation, CD3ζ+CD28 Retroviral vector	FDA 2017.10.18 EMA 2018.08.27	Adult patients with LBCL failing at least two other kinds of treatment (including r/r DLBCL, r/r PMBCL, high-grade BCL and DLBCL arising from FL) (Jacobson et al., 2020)	
Brexucabtagene autoleucel (Tecartus)	Kite pharma	CD19	Second generation, CD3ζ+CD28 Retroviral vector	FDA 2020.07.24 EMA 2020.12.17	Adult patients with r/r MCL Adults with r/r B-ALL (Tbakhi and Reagan, 2022)	
Lisocabtagene maraleucel (Breyanzi)	Juno Therapeutics/Bristol Myers Squibb	CD19	Second generation, CD3ζ+4-1BB Lentiviral vector	FDA 2021.02.05	Adult patients with r/r LBCL failing at least two other kinds of treatment (including r/r DLBCL, r/r PMBCL, high-grade BCL, Grade 3B FL) (Crees and Ghobadi, 2021)	
Idecabtagene Vicleucel (Abecma)	Bristol Myers Squibb	BCMA	Second generation, CD3ζ+4-1BB Lentiviral vector	FDA 2021.03.26 EMA 2021.08.19	Adult patients with r/r MM (Sharma et al., 2022)	
Relmacabtagene autoleucel (relma-cel)	JW Therapeutics	CD19	Second generation, CD3ζ+4-1BB Lentiviral vector	NMPA 2021.09.03	Adult patients with r/r DLBCL (Ying et al., 2021)	
Ciltacabtagene autoleucel (Carvykti)	Legend Biotech/Janssen Biotech	BCMA (consisting of two BCMA-binding domains)	Second generation, CD3ζ+4-1BB Lentiviral vector	FDA 2022.02.28	Adult patients with r/r MM (Berdeja et al., 2021)	

FDA, Food and Drug Administration; EMA, European Medicines Agency; NMPA, National Medical Products Administration; r/r B-ALL, relapsed or refractory B-cell acute lymphoblastic leukaemia; r/r DLBCL, relapsed or refractory diffuse large B-cell lymphoma; LBCL, large B-cell lymphoma; PMBCL, primary mediastinal B-cell lymphoma; BCL, B-cell lymphoma; FL, follicular lymphoma; MCL, mantle cell lymphoma; MM, multiple myeloma.

Compared with established radiotherapy and chemotherapy, the mechanism of adverse reactions related to CAR-T therapy is more complex and difficult to clarify. Cytokine release syndrome (CRS) and immune effector cell-associated neurotoxicity syndrome (ICANS) are the most common adverse events during CAR-T cell therapy (Fried et al., 2019; Dolladille et al., 2021). Previous clinical trials have suggested that during CAR-T cell treatment, the incidence of CRS was 57–93%, such that the severe form of CRS (≥ grade 3) had an incidence of 13–32%, the incidence of ICANS was 39–69%, and that of the severe form (≥ grade 3) was 11–41.5% (Neelapu et al., 2017; Schuster et al., 2017; Maude et al., 2018; Park et al., 2018; Santomasso et al., 2018; Cohen et al., 2019; Schuster et al., 2019; Nastoupil et al., 2020; Shalabi et al., 2020; Holtzman et al., 2021). In comparing two clinical studies, we observed that the incidence of CRS and ICANS after treatment with tisagenlecleucel was 58% and 12%, respectively, and was significantly lower than that of CRS (93%) and ICANS (64%) after the treatment with axicabtagene ciloleucel (Neelapu et al., 2017; Schuster et al., 2019). Severe CRS can lead to organ dysfunction; however, we lack options for excluding the influence of other mechanisms on these organ toxicities. Therefore, compared to conventional radiotherapy and chemotherapy, the mechanism of adverse reactions related to CAR-T therapy is more complex and challenging to elucidate.

In a retrospective pharmacovigilance study, Goldman et al. (2021) analyzed reports of 2,657 patients treated with axicabtagene-ciloleucel and tisagenlecleucel and suggested that the mortality rate of cardiovascular and pulmonary adverse events (CPAE) was 30.9%, which was significantly higher than that of CRS (17.4%). Moreover, among 546 patients with CPAE, 68.3% had concurrent CRS (Goldman et al., 2021). A combination of CRS with other organ system toxicity is common, and most CAR-T cell-induced adverse reactions (Table 2) could be managed if diagnosed early., However, the organ system toxicity of concurrent CRS is not easily recognized, thereby hindering the timely diagnosis and treatment. Thus, a comprehensive understanding of these adverse reactions their risk factors, and the management strategies for related adverse reactions are crucial in reducing mortality and improving recovery rates.

**TABLE 2:** Adverse reactions related to CAR-T cell therapy.

Adverse reaction	Main symptoms	Relationship with CRS	Characteristic
CRS	Fever; Hypotension; Hypoxia; DIC; Multi organ system toxicities	—	• Systemic inflammatory reaction caused by a large number of inflammatory factors
ICANS	Aphasia; Headache; Mild encephalopathy; Focal neurological Deficit; Tremor; Seizures; brain edema	CRS is one of the main inducers of ICANS, ICANS and CRS may occur simultaneously or not	• The breakdown of the BBB and capillary leakage lead to the entry of pro-inflammatory cytokines and CAR-T cells into the CSF to damage the CNS.
Cardiovascular toxicity	Hypotension; Sinus tachycardia; Increased serum troponin levels; Arrhythmia; Reduced LVEF; Cardiogenic shock; QT prolongation; Heart failure	CRS is one of the main inducers of cardiovascular toxicity, which can lead to serious direct and indirect cardiovascular complications	• Abnormal elevation of inflammatory cytokines IL-6, VWF, Ang-2, TNF-α and off-target cross-reaction of CAR-T cells to actin can lead to cardiovascular toxicity
Hematologic toxicity	Neutropenia; Thrombocytopenia; Leucopenia; Anemia; B-cell aplasia; Coagulopathy	Patients with severe CRS were more likely to develop late hematologic toxicity	• Neutropenia is closely related to infectious complications
			• B-cell aplasia is a common toxicity of anti-CD19 CAR-T therapy
HLH/MAS	Ferritin is extremely elevated; High fever; Hepatosplenomegaly; Hemocytopenia; Coagulopathy	HLH/MAS is a severe manifestation of CRS, so it is difficult to distinguish diagnosis of them	• The incidence of HLH/MAS is low, but its mortality is high and prognosis is poor
Skin toxicity	Rash; Dry skin; Purpura; Papules; Maculopapular; Urticarial rash; Bullous eruptions; Oral mucositis	CRS is one of the inducers of skin toxicity, and the reduced immune function induced by CRS may lead to skin infections in patients	• The clinical manifestations and mechanisms of skin toxicities are still poorly understood
			• Currently, there are no guidelines to diagnose and treat skin toxicity
Pulmonary toxicity	Respiratory failure	CRS is one of the main inducers of pulmonary toxicity	• The incidence of pulmonary toxicity is lower than that of CRS and ICANS.
			• There are definite clinical diagnostic indicators about pulmonary toxicity
Renal toxicity	Adrenal insufficiency; Electrolyte disorders; Kidney failure; Acidosis	CRS is one of the main inducers of renal toxicity	• The incidence of renal toxicity is lower than that of CRS and ICANS.
			• There are definite clinical diagnostic indicators about renal toxicity
			• Usually symptomatic treatment
Hepatotoxicity	Liver injury	CRS is one of the main inducers of hepatotoxicity	• The incidence of hepatotoxicity is lower than that of CRS and ICANS.
			• There are definite clinical diagnostic indicators about hepatotoxicity
Gastrointestinal toxicity	Diarrhea; Vomiting; Bleeding; Nausea	CRS is one of the main inducers of gastrointestinal toxicity	• The incidence of gastrointestinal toxicity is lower than that of CRS and ICANS.
			• There are definite clinical diagnostic indicators about gastrointestinal toxicity
			• Usually symptomatic treatment

CRS, cytokine release syndrome; ICANS, immune effector cell-associated neurotoxicity syndrome; DIC, disseminated intravascular coagulation; BBB, blood brain barrier; CSF, cerebrospinal fluid; CNS, central nervous system; LVEF, left ventricular ejection fraction; IL, interleukin; Ang-2, angiopoietin-2; VWF, von willebrand factor; TNF-α, tumor necrosis factor alpha; HLH/MAS, Hemophagocytic Lymphohistiocytosis/Macrophage Activation Syndrome.

## CAR-T cell therapy

**FIGURE 1:**
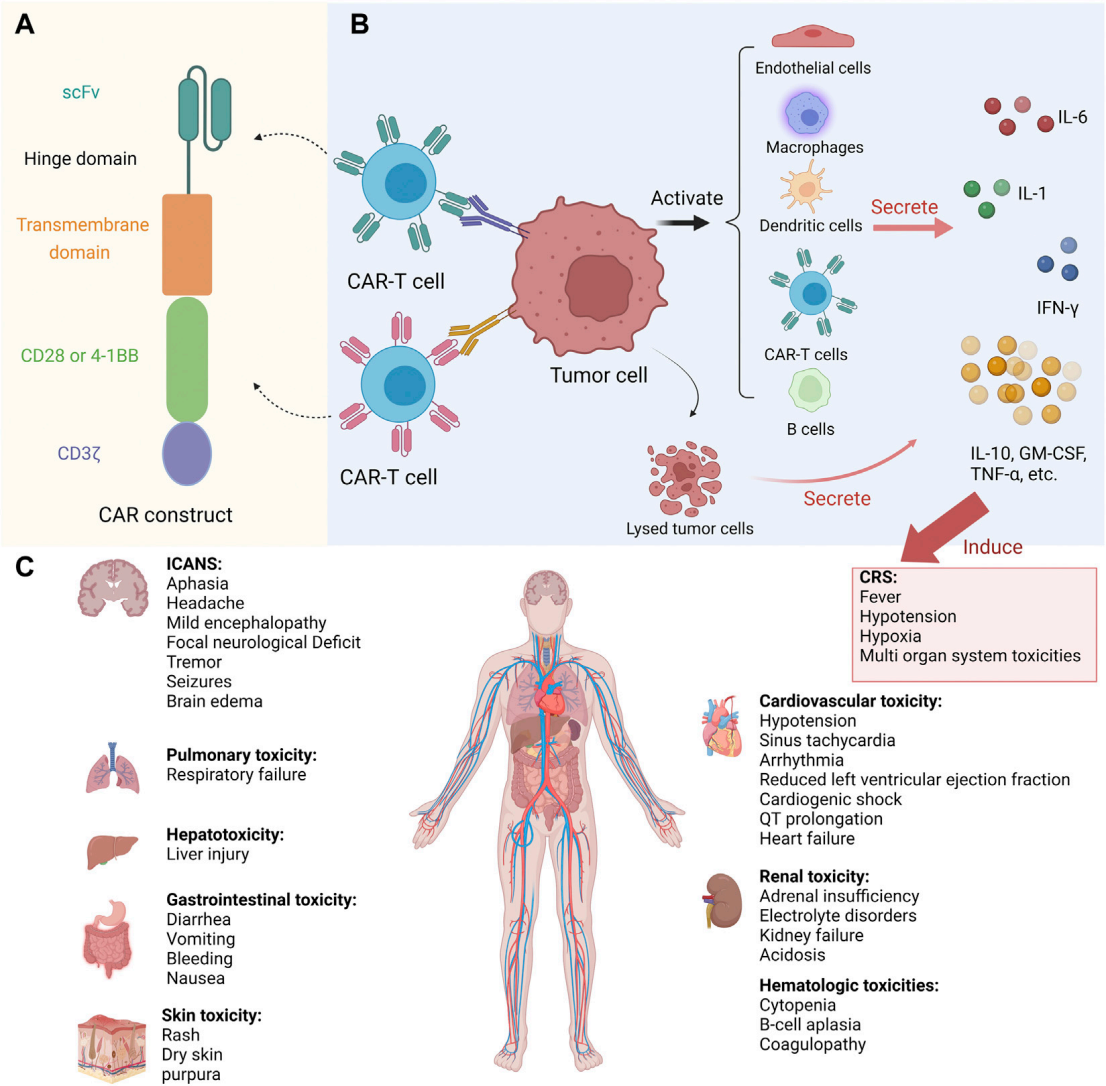
Toxicities during CAR-T therapy. **(A)** The structure of CAR. **(B)** Pathogenesis of CRS. **(C)** Organ systemic toxicities induced by CRS. Abbreviations: CAR, chimeric antigen receptor; CRS, cytokine release syndrome; ICANS, immune effector cell-associated neurotoxicity syndrome; scFv, single-chain variable fragment; IL, interleukin; IFN-γ, interferon gamma; GM-CSF, granulocyte macrophage colony-stimulating factor; TNF-α, tumor necrosis factor alpha. This figure created with BioRender.com.

The primary process of autologous CAR-T therapy is to first collect T cells, then genetically modify them to identify tumor antigens and amplify CAR-T cells, and finally introduce lymphodepletion chemotherapy prior to infusion of CAR-T cells back into the patient (Subklewe et al., 2019; Hong et al., 2020). Notably, lymphodepletion chemotherapy causes events such as infection and cytopenia. Currently, the marketed target antigens of CAR-T products include CD19 and BCMA. Numerous target antigens, including CD22, CD33, CD70, CD123, CD138, CD171, HER2, EGFR, B7-H3, claudin 6, gp120, GPRC5D, PSMA, and mesothelin, have been studied (Larson, Maus; Johnson and June, 2017; Smith et al., 2019; Sauer et al., 2021). Clinical studies on these targets are promising for CAR-T cell therapy in treating of r/r advanced solid tumors, autoimmune diseases, and acquired immunodeficiency syndrome (AIDS) (Rust et al., 2020; Mougiakakos et al., 2021; Totzeck et al., 2022). CAR comprises four domains (Figure 1A); extracellular antigen recognition, hinge, transmembrane connecting, and intracellular activating domains (Neelapu et al., 2018a; Hong et al., 2020; Larson, Maus; Nusbaum et al., 2021). The extracellular part consists of a single-chain variable fragment (scFv) of a monoclonal antibody (responsible for recognizing and binding tumor antigens) and a hinge region that acts as a linker, whereas the intracellular part consists of signal transduction domains and single or multiple T cell costimulatory domains (Badieyan and Hoseini, 2018; Stoiber et al., 2019). The intracellular domain of the first-generation CAR is composed of CD3ζ, whereas that of the second-generation CAR is composed of CD3ζ and a costimulatory domain (CD28 or 4-1BB), and that of the third-generation CAR is composed of CD3ζ and two costimulatory domains (CD28 and 4-1BB) (Kosti et al., 2018; Mochel et al., 2019). The expansion and persistence of second- and third-generation CAR-T cells with costimulatory domains are significantly improved compared to that of first-generation CAR-T cells (Imai et al., 2004; Savoldo et al., 2011). The marketed CAR-T therapies all involve second-generation CARs, where the intracellular domains of axicabtagene ciloleucel and brexucabtagene autoleucel consist of CD3ζ and the costimulatory domain CD28 (Reagan, Friedberg). The intracellular domain of the other five CAR-T therapies consist of CD3ζ and the costimulatory domain 4-1BB (Table 1).

CAR-T manufacturing generally takes 2–4 weeks and may extend to 3–6 weeks due to the turnaround and transportation time to the final infusion into the patient (Freyer and Porter, 2020). The turnaround time for CAR-T manufacturing/delivery varies with the products and the physical condition of patients. The primary sources of T cells in CAR-T cell immunotherapy are allogeneic and autologous. Allogeneic CAR-T cell therapy has a higher incidence of graft *versus* host disease (GVHD) than autologous CAR-T cell therapy; however, it is more beneficial in mass production, timely supply to cancer patients, and low production cost. It is currently the most promising method for the clinical application of CAR-T batches. However, the current CAR-T cell therapy mainly employs autologous T cells, which have a long production cycle and are expensive (Mahadeo et al., 2019).

## The main adverse reactions of CAR-T cell therapy

### Cytokine release syndrome – Diagnosis and treatment

CRS is the most common adverse reaction to CAR-T cell infusion. It is mainly a systemic inflammatory reaction caused by a large number of inflammatory factors released by activated immune cells (T cells, macrophages, B cells, monocytes, natural killer cells, and dendritic cells) and endothelial cells (Figure 1B) (Dal'bo et al., 2020; Neelapu et al., 2018a; Giavridis et al., 2018; Ganatra et al., 2019b). Following CAR-T cell infusion, the onset of CRS ranges from hours to days as T cells expand (Maude et al., 2014). The timing of CRS occurrence is closely related to the structure of CAR. For example, patients treated with anti-CD19-CD28-CD3ζ CAR typically develop CRS earlier than those treated with anti-CD19-4-1BB-CD3ζ CAR (Neelapu et al., 2018a).

Common signs of CRS are fever (≥38°C), hypotension (systolic blood pressure <90 mmHg), and hypoxia (oxygen saturation <90%) (Neelapu et al., 2018a). Severe CRS can induce disseminated intravascular coagulation (DIC), multiple organ toxicity (Figure 1C) (adult respiratory distress syndrome, ICANS, cardiac dysfunction, cytopenia), and even death (Neelapu et al., 2018a; Ganatra et al., 2019b; Sterner and Sterner, 2021). Risk factors for severe CRS include patient-related factors (B-ALL diagnosis, high tumor burden, baseline thrombocytopenia, and endothelial activation), tumor-related factors (B-ALL diagnosis), and treatment-related factors (high number of infused CAR-T cells, high peak of CAR-T cell expansion, CD28 co-stimulatory, high-intensity lymphodepletion regimens) (Jin et al., 2018; Ganatra et al., 2019b; Zheng et al., 2020; Schubert et al., 2021).

IL-6 produced by human circulating monocytes is a key cytokine that leads to CRS in CAR-T therapy (Norelli et al., 2018). The FDA approved tocilizumab (an IL-6 receptor antagonist) for treating CAR-T cell-induced CRS in 2017. The recommended therapeutic dose of tocilizumab is 4–8 mg/kg (maximum 800 mg) (Brudno and Kochenderfer, 2016; Le et al., 2018). Thus, tocilizumab is not recommended for inflammation induced by infection, neutropenic sepsis, or tumor lysis syndrome (TLS). CRS must be confirmed before tocilizumab treatment. ZUMA-1 cohort 4 and 6 studies have demonstrated that prior use of corticosteroids and/or tocilizumab and prophylactic corticosteroids may reduce the incidence of ≥ grade 3 CRS and ICANS (Oluwole et al., 2021; Topp et al., 2021). Regarding the early identification and prediction of CRS, Teachey et al. (2016) further screened three specific markers from 24 biomarkers for predicting severe CRS. Differences by age were found, with predicted biomarkers gp130, IFN-γ, and IL-1RA for adults and IFN-γ, IL-13, and MIP-1α in children (Teachey et al., 2016). It is expensive to predict whether a patient is likely to develop severe CRS based on the measurement of multiple cytokines. Pennisi et al. (2021) found that the modified endothelial activation and stress index (EASIX) score (lactate dehydrogenase [LDH; U/L] × C-reactive protein [CRP; mg/dl]/platelets [PLTs; 10^9^ cells/L]) is the most clinically relevant formula for predicting severe CRS and ICANS. Hay et al. (2017) designed a simple two-step algorithm to predict grade ≥4 CRS; they first checked whether the patient had a fever ≥38.9°C within 36 h of CAR-T infusion and then performed serum MCP-1. These methods facilitate the prediction of severe toxicity during CAR-T therapy.

The consensus criteria for grading (Table 3) and management (Table 4) of CRS are invaluable for treating this particular toxicity. Historically, there are numerous grading systems for CRS, but clinicians typically use the consensus American Society of Transplantation and Cellular Therapy (ASTCT) guidelines (Pennisi et al., 2020). ASTCT (Lee et al., 2019) defines CRS as “a supraphysiological response following any immune therapy that results in the activation or engagement of endogenous or infused T-cells and/or other immune effector cells. The onset of symptoms can be progressive, must include fever at the onset, and may include hypotension, capillary leak (hypoxia), and end-organ dysfunction.” In addition, Santomasso et al. (2021) systematically reviewed the evidence of immune-related adverse events in patients treated with CAR-T cells published from 2017 to 2021 and developed ASCO guidelines in conjunction with a multidisciplinary team (consisting of medical oncology, neurology, hematology, emergency medicine, nursing). However, owing to the lack of high-quality evidence, recommendations are based only on the consensus of experts. The CRS grading criteria in the ASCO guidelines were formulated based on the ASTCT consensus, as shown in Table 3. Regarding CRS treatment (Table 4), all guidelines involve supportive care (treatment of fever, hypotension, and hypoxia). Tocilizumab is the first-line drug used for the treatment of CRS. Corticosteroids (dexamethasone or methylprednisolone) should be added when tocilizumab fails to effectively control CRS or when CRS worsens. However, consensuses differ on varying dose regimens in treating patients with CRS Furthermore, in order to reduce the harm of severe CRS to patients, some prophylactic drugs (such as dexamethasone, anakinra, itacitinib, tocilizumab) are used in patients receiving CAR-T therapy. The specific clinical trials are shown in Table 5.

**TABLE 3:** Grading of CRS.

CRS grading system	CTCAE version 5.0 (National Cancer Institute, 2017)	Lee criteria (Lee et al., 2014)	CARTOX criteria (Neelapu et al., 2018a)	ASTCT consensus criteria (Lee et al., 2019)	ASCO guideline (Santomasso et al., 2021)
Grade 1	• Fever (≥38.0°C)	Symptoms are not life-threatening and require symptomatic treatment only (e.g., fever, nausea, fatigue, headache, myalgias, malaise)	• Fever (≥38.0°C)	• Fever (≥38.0°C)	• Fever (≥38.0°C) not attributable to any other cause
	• And/or constitutional symptoms		• No hypotension	• No hypotension	• No hypotension
			• No hypoxia	• No hypoxia	• No hypoxia
			• And/or grade 1 organ toxicities (CTCAEv4.03)		
Grade 2	• Fever (≥38.0°C)	Symptoms require and respond to moderate intervention	• Fever (≥38.0°C)	• Fever (≥38.0°C)	• Fever (≥38.0°C) not attributable to any other cause
	• Hypotension (responds to fluids)	• Hypotension (responds to IV fluids or low dose of one vasopressor)	• Hypotension (Responds to IV fluids or low-dose vasopressors)	• And hypotension not requiring vasopressors	• And hypotension not requiring vasopressors
	• hypoxia (FiO2 <40%)	• Hypoxia (FiO2 <40%)	• Or hypoxia (FiO2 <40%)	• And/or hypoxia requiring low-flow nasal cannula (≤6 L/min)	• And/or hypoxia requiring low-flow nasal cannula (≤6 L/min) or blowby
		• Grade 2 organ toxicity (CTCAEv4.03)	• Or grade 2 organ toxicities (CTCAEv4.03)		
Grade 3	• Fever (≥38.0°C)	Symptoms require and respond to aggressive intervention	• Fever (≥38.0°C)	• fever (≥38.0°C)	• Fever (≥38.0°C) not attributable to any other cause
	• Hypotension (needs one vasopressors)	• Hypotension (responds to high-dose or multiple vasopressors)	• Hypotension (needs high-dose or multiple vasopressors)	• And hypotension requiring one vasopressor ± vasopressin	• And hypotension requiring one vasopressor ± vasopressin
	• hypoxia (FiO2 ≥40%)	• hypoxia (FiO2 ≥40%)	• Or hypoxia (FiO2 ≥40%)	• And/or hypoxia requiring high-flow nasal cannula (>6 L/min), facemask, non-rebreather mask, or venturi mask	• And/or hypoxia requiring high-flow nasal cannula, facemask, non-rebreather mask, or venturi mask
		• Grade 3 organ toxicity or grade 4 transaminitis (CTCAEv4.03)	• Or grade 3 organ toxicity or grade 4 transaminitis (CTCAEv4.03)		
Grade 4	• Fever (≥38.0°C)	Life-threatening symptoms	• Fever (≥38.0°C)	• fever (≥38.0°C)	• Fever (≥38.0°C) not attributable to any other cause
	• Life-threatening consequences; urgent intervention needed	• Hypoxia (needing ventilator support)	• Hypotension (Life-threatening)	• And hypotension requiring multiple vasopressors (excluding vasopressin)	• And hypotension requiring multiple vasopressors (excluding vasopressin)
		• Grade 4 organ toxicity except grade 4 transaminitis (CTCAEv4.03)	• Or hypoxia (needing ventilator support)	• And/or hypoxia requiring positive pressure (e.g., CPAP, BiPAP, intubation and mechanical ventilation)	• And/or hypoxia requiring positive pressure (e.g., CPAP, BiPAP, intubation and mechanical ventilation)
			• Or grade 4 organ toxicity except grade 4 transaminitis (CTCAEv4.03)		
Grade 5	Death	Death	—	death due to CRS	—

Hypotension, Systolic blood pressure <90 mmHg; Hypoxia, Needing oxygen for SaO2 >90%.

High-dose vasopressors (all doses are required for ≥3 h) (Lee et al., 2014) are defined as any of the following: noradrenaline ≥20 μg/kg/min; dopamine ≥10 μg/kg/min; phenylephrine ≥200 μg/kg/min; adrenaline ≥10 μg/kg/min; if on vasopressin, vasopressin + noradrenaline equivalent of ≥10 μg/kg/min; if on combination vasopressors (not including vasopressin), noradrenaline equivalent of ≥20 μg/kg/min. VASST Trial vasopressor equivalent equation: norepinephrine equivalent dose = [norepinephrine (μg/min)] + [dopamine (μg/kg/min) ÷ 2] + [epinephrine (μg/min)] + [phenylephrine (μg/min) ÷10].

CRS, cytokine release syndrome; CTCAE, Common Terminology Criteria for Adverse Events; FiO2, fraction of inspired oxygen; IV, intravenous; CTCAE, Common Terminology Criteria for Adverse Events; CARTOX, CAR-T cell therapy associated toxicity; ASTCT, American Society for Transplantation and Cellular Therapy; CPAP, continuous positive airway pressure; BiPAP, Bilevel positive airway pressure; ASCO, American Society of Clinical Oncology.

**TABLE 4:** Management of CRS.

CRS management system	Lee criteria (Lee et al., 2014)	CARTOX criteria (Neelapu et al., 2018a)	ASTCT consensus criteria (Neelapu, 2019)	ASCO guideline (Santomasso et al., 2021)
Grade 1	• Vigilant supportive care (treat fever and neutropenia if present, antipyretics, analgesics as needed)	• Fever: Acetaminophen and hypothermia blanket; Consider tocilizumab 8 mg/kg IV or siltuximab 11 mg/kg IV for persistent (lasting >3 days) and refractory fever	•Antipyretics and IV hydration	• Supportive care with antipyretics, IV hydration, and symptomatic management of organ toxicities and constitutional symptoms
	• Assess for infection	• Organ toxicity: Symptomatic management	• Diagnostic work-up to rule out infection	• Consider empiric broad-spectrum antibiotics if neutropenic
	• Monitor fluid balance	• Empiric broad-spectrum antibiotics and filgrastim if neutropenic	• Consider growth factors and antibiotics if neutropenic	• If neutropenia, consider empiric broad-spectrum antibiotics and G-CSF (GM-CSF is not recommended)
		• Maintenance IV fluids for hydration		• In patients with persistent (>3 days) or refractory fever, consider managing as per grade 2
Grade 2	• Maintenance of adequate hydration and blood pressure	• Fever: manage fever as in grade 1 CRS	• Supportive care as in grade 1	• Supportive care as per grade 1
	• Vigilant supportive care (monitor cardiac and other organ function closely), if the patient doesn’t have extensive co-morbidities or older age	• Hypotension: IV fluid bolus of 500–1,000 ml of normal saline; Second IV fluid bolus if pressure remains <90 mmHg; Tocilizumab or siltuximab for the hypotension refractory to fluid boluses (tocilizumab can be repeated after 6 h); If hypotension persists, start vasopressors, consider transfer to ICU), dexamethasone (10 mg q6h, IV)	• IV fluid boluses and/or supplemental oxygen	• Administer tocilizumab (8 mg/kg, IV); Repeat q8h if no improvement in signs and symptoms of CRS; Limit to a maximum of three doses in a 24 h period, with a maximum of four doses total
	• Tocilizumab (adults 4 mg/kg, children 8 mg/kg) ± corticosteroids (methylprednisolone 2 mg/kg/day, dexamethasone 0.5 mg/kg maximum 10 mg/dose), if the patient has extensive co-morbidities or older age	• Hypoxia: supplemental oxygen; Tocilizumab or siltuiximab ± corticosteroids and supportive care	• Tocilizumab ± dexamethasone or its equivalent of methylprednisolone	• In patients with hypotension that persists after two fluid boluses and after one to two doses of tocilizumab, may consider dexamethasone (10 mg q12h, IV) for one to two doses and then reassess
		• Organ toxicity: symptomatic management of organ toxicities, as per standard guidelines; Tocilizumab or siltuiximab ± corticosteroids and supportive care		• Manage per grade 3 if no improvement within 24 h of starting tocilizumab
Grade 3	• Maintenance of adequate hydration and blood pressure	• Fever: manage fever as in grade 1 CRS	• Supportive care as in grade 1	• Supportive care as per grade 2 and include vasopressors as needed
	• Vigilant supportive care	• Hypotension: IV fluid bolus, tocilizumab and siltuximab as recommended for grade 2 CRS; Increase dexamethasone to 20 mg q6h IV, if refractory; Transfer to ICU, obtain echocardiogram, and perform haemodynamic monitoring	• Consider monitoring in intensive care unit	• Tocilizumab as per grade 2 if maximum dose is not reached within 24 h period plus dexamethasone (10 mg q6h, IV) and taper once symptoms improve
	• Tocilizumab (adults 4 mg/kg, children 8 mg/kg) ± corticosteroids (methylprednisolone 2 mg/kg/day, dexamethasone 0.5 mg/kg maximum 10 mg/dose)	• Hypoxia: supplemental oxygen including high-flow oxygen delivery and non-invasive positive pressure ventilation; Tocilizumab or siltuximab + corticosteroids	• Vasopressor support and/or supplemental oxygen	• If echocardiogram was not already performed, obtain ECHO to assess cardiac function and conduct hemodynamic monitoring
		• Organ toxicity: symptomatic management of organ toxicities, as per standard guidelines; Tocilizumab or siltuximab + corticosteroids	• Tocilizumab + dexamethasone (10–20 mg q6h, IV) or its equivalent of methylprednisolone	• If refractory, manage as per grade 4
				• Admit patient to ICU
Grade 4	• maintenance of adequate hydration and blood pressure	• Fever: manage fever as in grade 1 CRS	• Supportive care as in grade 1	• Supportive care as per grade 3 plus mechanical ventilation as needed
	• Vigilant supportive care	• Hypotension: manage hypotension as in grade 3 CRS; Methylprednisolone (1 g/day, IV)	• Monitoring in intensive care unit	• Tocilizumab as per grade 2 if maximum dose is not reached within 24 h period; Initiate high-dose methylprednisolone (500 mg q12h, IV) for 3 days, followed by 250 mg IV q12h for 2 days, 125 mg IV q12h for 2 days, and 60 mg IV q12h until CRS improvement to grade 1
	• Tocilizumab (adults 4 mg/kg, children 8 mg/kg) ± corticosteroids (methylprednisolone 2 mg/kg/day, dexamethasone 0.5 mg/kg maximum 10 mg/dose)	• Hypoxia: mechanical ventilation; Tocilizumab or siltuximab + corticosteroids	• Vasopressor support and/or supplemental oxygen *via* positive pressure ventilation	• If not improving, consider methylprednisolone (1g, IV) 2 times a day or alternate therapy
		• Organ toxicity: symptomatic management of organ toxicities, as per standard guidelines; Tocilizumab or siltuximab + corticosteroids	• Tocilizumab + methylprednisolone 1 g/day	
			

Tocilizumab IV over 1 h, Maximum amount of tocilizumab per dose is 800 mg.

CRS, cytokine release syndrome; CARTOX, CAR-T cell therapy associated toxicity; IV, intravenous; ICU, intensive-care unit; q6h, every 6 hours; q8h, every 8 hours; q12h, every 12 hours; ASTCT, American Society for Transplantation and Cellular Therapy; ASCO, American Society of Clinical Oncology; G-CSF, granulocyte-colony stimulating factor; GM-CSF, granulocyte-macrophage colony-stimulating factor; ECHO, echocardiography.

**TABLE 5:** Current interventional clinical trials aiming to reduce CAR-T specific toxicities.

Name	Clinical trials	Specific toxicities	Prophylactic drug	Recruitment status
Axicabtagene ciloleucel	NCT05459571	CRS ICANS	Dexamethasone: dexamethasone (10 mg, orally or IV) before CAR-T cell infusion	Recruiting
Axicabtagene ciloleucel	NCT04314843	ICANS	Lenzilumab: sequenced therapy of lenzilumab and axicabtagene ciloleucel on Day 0	Terminated (Development program terminated.)
Axicabtagene ciloleucel	NCT04150913	CRS ICANS	Anakinra: anakinra (dosage per protocol, SC) on days 0–6	Recruiting
Axicabtagene ciloleucel	NCT04514029	ICANS	Simvastatin: simvastatin (40 mg/day, orally) will be started at least 5 days prior to apheresis and will be continued until day +30 after infusion. Dexamethasone: intrathecal dexamethasone 8 mg on days −1, +6, +13 ( ± 2 days)	Recruiting
Axicabtagene ciloleucel	NCT04432506	CRS ICANS	Anakinra: anakinra SC on days 0–6	Active, not recruiting
Axicabtagene ciloleucel	NCT03954106	ICANS	Defibrotide: defibrotide 6.25 mg/kg/dose once daily as a single dose on CAR-T Day −5, −4, and −3 before lymphodepletion, then every 6 h daily for 8 days (CAR-T Day 0 to Day 7)	Terminated (Primary endpoint would unlikely to be met based on the unplanned interim assessment on the first 20 efficacy evaluable patients.)
Axicabtagene ciloleucel	NCT04205838	ICANS	Anakinra: anakinra SC every 6–12 h for 12–36 doses over 9 days	Suspended (funding)
Axicabtagene ciloleucel	NCT04071366	CRS	Itacitinib: itacitinib (200 mg/day, orally) for 30 days or itacitinib (200 mg bid, orally) for 30 days	Recruiting
Axicabtagene ciloleucel	NCT02348216	CRS ICANS	Cohort 3	Active, not recruiting
			Levetiracetam: levetiracetam (750 mg orally or IV, BID) starting on Day 0	
			Tocilizumab: tocilizumab (8 mg/kg IV over 1 h [not to exceed 800 mg]) on Day 2	
			Cohort 4	
			Corticosteroids: dexamethasone or methylprednisolone. Tocilizumab: tocilizumab (8 mg/kg IV over 1 h [not to exceed 800 mg] at lower grades of toxicity)	
			Levetiracetam: levetiracetam (750 mg orally or IV, BID) starting on Day 0	
			Cohort 5	
			Levetiracetam: levetiracetam (750 mg orally or IV, BID) starting on Day 0	
			Cohort 6	
			Corticosteroids: dexamethasone prior to axicabtagene ciloleucel infusion on Day 0, Day 1 and Day 2	
			Tocilizumab: tocilizumab at lower grades of toxicity	
			Levetiracetam: levetiracetam (750 mg orally or IV, BID) starting on Day 0	
Lisocabtagene maraleucel	NCT04359784	CRS ICANS	Anakinra: anakinra SC daily on days 0–13	Recruiting

CRS, cytokine release syndrome; ICANS, immune effector cell-associated neurotoxicity syndrome; IV, intravenous; SC, Subcutaneous Injections.

### ICANS

ICANS, also known as neurotoxicity or CAR-T-cell-related encephalopathy syndrome (CRES), is another unique toxicity of CAR-T therapy, but is less likely to occur than CRS (Neelapu et al., 2018a; Miao et al., 2021). It was defined as any new and well-defined neurological symptom that occurred within 60 days of CAR-T cell infusion and was attributable to the infusion (Gofshteyn et al., 2018). Common signs and symptoms of ICANS include aphasia, headache, mild encephalopathy, focal neurological deficits, tremors, seizures, and rarely, fatal cerebral edema (Hirayama and Turtle, 2019; Ragoonanan et al., 2021). In addition, Van Oekelen et al. (2021) reported rare neurocognitive and hypokinetic movement disorders with Parkinsonian tendencies in patients using anti-BCMA CAR-T therapy. The time to ICANS onset during CAR-T therapy ranged from 0 to 19 days (Gust et al., 2017; Santomasso et al., 2018; Schuster et al., 2019; Holtzman et al., 2021). ICANS and CRS can occur simultaneously or not. In most cases, ICANS occurs after the complete resolution of CRS; if CRS does not occur before ICANS, ICANS is usually mild (Santomasso et al., 2018; Freyer and Porter, 2020; Neill et al., 2020). Santomasso et al. (2018) studied 53 patients treated with 19-28z CAR-T cells and found that all patients with neurological symptoms developed at least grade 1 CRS with fever. Gust et al. (2017) studied neurological adverse reactions in 133 patients infused with CAR-T cells and observed that ≥ grade 3 neurotoxicity was often accompanied by more severe CRS and endothelial dysfunction (diffuse intravascular coagulation, capillary leakage, and blood-brain barrier disruption). CRS is closely related to severe ICANS, but not all cases of ICANS are accompanied by CRS. Most pathophysiological studies of ICANS are based on imaging findings in patients with severe or fatal neurotoxicity, such as cerebral edema. Tumor-associated non-occlusive thrombosis, petechial hemorrhages, pontine infarcts, and non-specific white matter changes were found in 28% of patients with ICANS compared to baseline brain MRI before CAR-T cell treatment (Holtzman et al., 2021). However, further investigation is required to ascertain whether these pathophysiological mechanisms apply to mild reversible neurotoxicity.

#### Pathogenesis of ICANS

**FIGURE 2:**
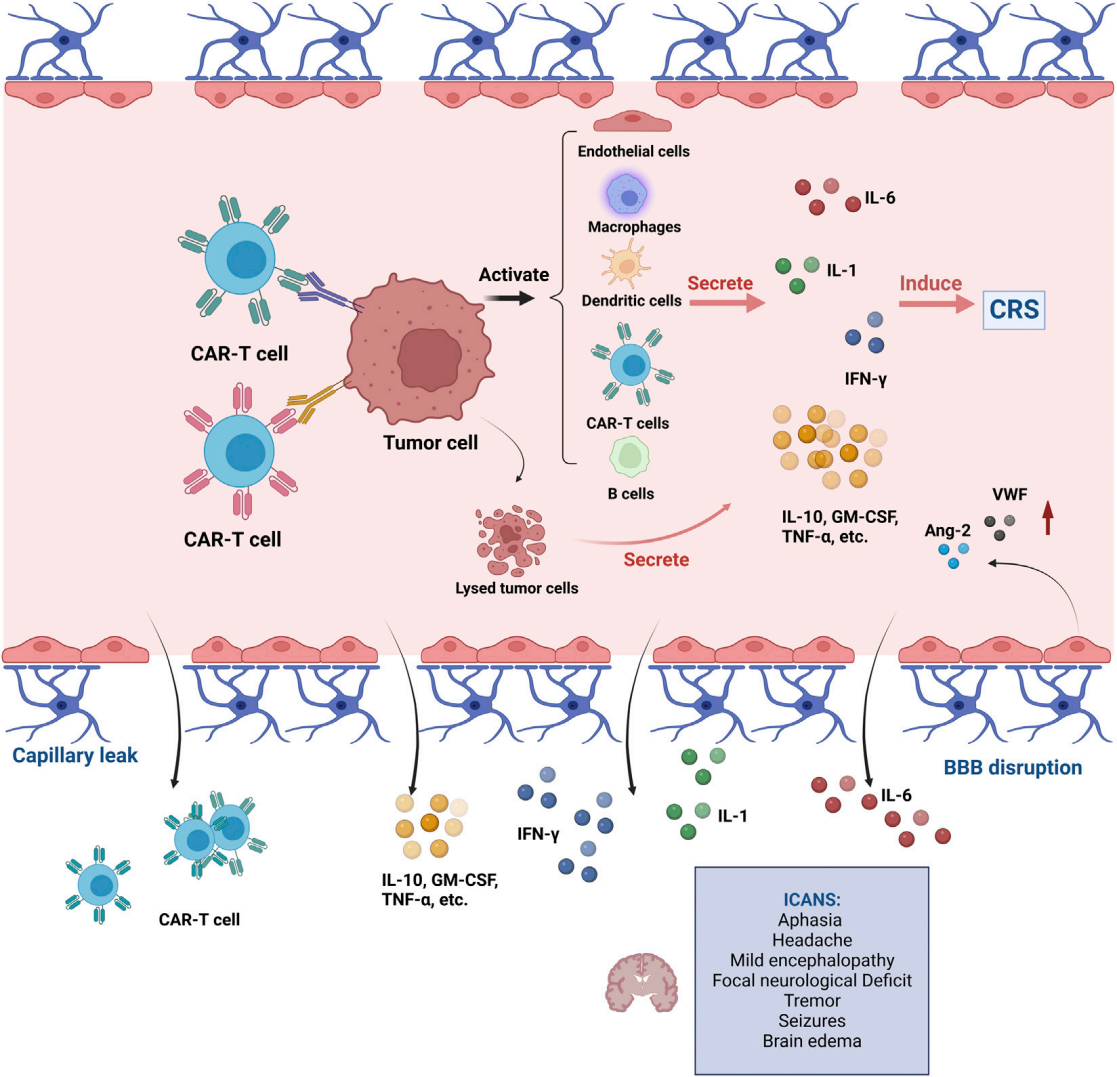
Pathogenesis of ICANS during CAR-T therapy. Abbreviations: CAR, chimeric antigen receptor; CRS, cytokine release syndrome; ICANS, immune effector cell-associated neurotoxicity syndrome; IL, interleukin; IFN-γ, interferon gamma; GM-CSF, granulocyte macrophage colony-stimulating factor; TNF-α, tumor necrosis factor alpha; BBB, blood brain barrier. This figure created with BioRender.com.

CRS is one of the main inducers of ICANS, and the pathogenesis of both is similar but not entirely consistent. The potential pathogenesis of ICANS may be related to the following mechanisms (Figure 2): *1*) blood-brain barrier breakdown, increased central nervous system (CNS) vascular permeability, followed by diffusion of inflammatory cytokines (IL-6, IL-15, and IFN-γ) into the CNS, ultimately exacerbating neurotoxicity (Gust et al., 2017). *2*) Direct CNS toxicity of CAR-T cells in cerebrospinal fluid (CSF). Studies have shown that patients with neurotoxicity have a significantly higher number of CAR-T cells in the CSF than those without neurotoxicity (Lee et al., 2015). *3*) CRS-induced hypoxemia can cause ICANS. *4*) CNS resident cells (endothelial, pericyte, microglia, astrocytes) secrete ICANS-related cytokines after CAR-T infusion. CSF levels of S100 calcium-binding protein B and glial fibrillary acidic protein increase during neurotoxicity, resulting in astrocyte damage (Gust et al., 2019; Gust et al., 2020).

#### Risk factors for ICANS

Similar but not identical to the risk factors of CRS, the risk factors of ICANS mainly include: *1*) patients with neurologic comorbidities or other high disease burden before CAR-T therapy (Gust et al., 2017; Santomasso et al., 2018). *2*) Fever ≥38.9°C within 36 h after CAR-T cell infusion (Gust et al., 2017). *3*) Lymphocyte depletion therapy with fludarabine and cyclophosphamide, high tumor burden during CAR-T infusion, high-dose infusion of CAR-T cells, the high peak of CAR-T cell expansion, pretreatment thrombocytopenia, and endothelial activation (Kochenderfer et al., 2017; Nastoupil et al., 2020; Holtzman et al., 2021; Schubert et al., 2021). *4*) Comparing the two studies, it was found that the incidence of epilepsy (8%) of CAR-T cells containing the 4-1BB costimulatory domain was lower than that of CAR-T cells containing the CD28 costimulatory domain (48%) (Gust et al., 2017; Santomasso et al., 2018).

#### Possible cytokine predictors of ICANS

Different from the definite role of IL-6 in CRS, no single cytokine is know to affect ICANS; therefore, predicting severe ICANS with many cytokines may be a new promising direction. Santomasso et al. (2018) predicted severe neurotoxicity through changes in multiple cytokines and found that patients with low IL-15 (<50 pg/ml) or high EGF (>120 pg/ml) had a lower risk of severe neurotoxicity; patients with high IL-15, low EGF, and low IL-10 (<200 pg/ml) were at moderate risk; and patients with high IL-15, low EGF, and high IL-10 levels were at high risk of severe neurotoxicity. Kochenderfer et al. (2017) also found that patients with ICANS (≥ grade 3) had higher peak levels of serum IL-10 and IL-15. Other studies have revealed that elevated fibrinogen and ferritin levels during early CAR-T cell infusion, or serum IL-6 ≥ 16 pg/ml and MCP-1 ≥ 1343.5 pg/ml within 36 h after CAR-T cell infusion, may predict high-risk patients with ICANS (Gust et al., 2017; Holtzman et al., 2021).

Gust et al. (2020) analyzed eight studies and reported that serum concentrations of IL-6, IL-10, GM-CSF, IFN-γ, IL-15, IL-2, GzB, IL-2Rα, IL-1RA, and CXCL10 positive correlated with the onset of ICANS. IFN-γ, GM-CSF, IL-6, IL-10, and IL-15 are closely related to ICANS and are potential predictors of ICANS. This paper also summarized 10 studies (Table 6) and found that ICANS was closely related to increased cytokines such as IL-15, IL-10, IFN-γ, and IL-6. However, confirming the relationship between most cell biomarkers and ICANS is challenging owing to the limited reports on their association and several interference factors. For example, Faramand et al. (2020) and Gust et al. (2017) found that ICANS was associated with an increase in the serum biomarkers, angiopoietin-2 (Ang-2) and von Willebrand factor (VWF) related to endothelial activation. However, another study demonstrated that serum VWF, VEGF-A, Ang-1, and Ang-2 levels are not associated with neurotoxicity (Gust et al., 2019).

**TABLE 6:** Cytokines related to ICANS.

Researchers	CAR-T products	Number of patients	Cancer type	Relative cytokines
Gofshteyn et al. (2018)	Tisagenlecleucel (CTL09)	51	B-cell ALL (*n* = 50)	IL-2, sIL-4R, HGF, IL-15, sTNFR-1
			T-cell ALL (*n* = 1)	
Cohen et al. (2019)	CART-BCMA	25	r/r MM	IL-1RA, IFN-γ, MIP-1α, IL-6
Gust et al. (2019)	SCRI-CAR19v1	43	r/r B-ALL	GM-CSF, TNF-α, MIP-1α, IFN-γ, IL-6, IL-10, GzB
Santomasso et al. (2018)	19-28z CAR-T	53	r/r B-ALL	IL-1α, IL-2, IL-3, IL-5, IL-6, IL-10, IL-15, IP-10, INF-γ, G-CSF, GM-CSF, MCP-1, EGF (decrease)
Shalabi et al. (2018)	Anti-CD22 CAR-T	22	r/r B-ALL (*n* = 21)	TNF-α, IL-6, IL-8, IL-15, IL-2, IL-10, IL-13, GM-CSF, IFN-γ, MIP-α
			r/r DLBCL (*n* = 1)	
Hay et al. (2017)	Anti-CD19 CAR-T	133	r/r B-ALL (*n* = 47)	IL-8, IL-6, IL-10, IL-15, IFN-γ, TNFRp55, MCP-1, MIP-1β
			r/r CLL (*n* = 24)	
			r/r NHL (*n* = 62)	
Neelapu et al. (2017)	axicabtagene ciloleucel	111	DLBCL (*n* = 81)	IL-1RA, IL-2Rα, IL-2, IL-6, IL-8, IL-10, IL-15, IFN-g, GzB, GM-CSF, ferritin
			PMBCL or TFL (*n* = 30)	
Faramand et al. (2020)	axicabtagene ciloleucel	75	LBCL or indolent lymphoma	IL-6, Ang-2/Ang-1 ratio, Ang-2, IL-15, IFN-γ, Ang-1 (decrease), ferritin
Gust et al. (2017)	Anti-CD19 CAR-T	133	B-ALL (*n* = 47)	IL-6, IFN-γ, TNF-α, Ang-2, VWF
			NHL (*n* = 62)	
			CLL (*n* = 24)	
Kochenderfer et al. (2017)	Anti-CD19 CAR-T	22	DLBCL (n = 19)	GzB, IL-10, IL-15, IFN-γ
			FL (n = 2)	
			MCL (n = 1)	
Park et al. (2017)	19-28z CAR-T	51	r/r B-ALL	GM-CSF, IFN-γ, IL-15, IL-5, IL-10, IL-2, ferritin

ALL, acute lymphoblastic leukaemia; r/r MM, relapsed/refractory multiple myeloma; r/r B-ALL, relapsed or refractory B-cell acute lymphoblastic leukaemia; r/r DLBCL, relapsed or refractory diffuse large B-cell lymphoma; r/r CLL, relapsed or refractory chronic lymphocytic leukemia; r/r NHL, relapsed or refractory non-hodgkin lymphoma; PMBCL, primary mediastinal B-cell lymphoma; TFL, transformed follicular lymphoma; LBCL, Large B Cell Lymphoma; FL, follicular lymphoma; MCL, mantle cell lymphoma; IL, interleukin; sIL, soluble interleukin; HGF, hepatocyte growth factor; sTNFR-1, soluble tumor necrosis factor 1; IFN-γ, interferon gamma; MIP-1α, macrophage inflammatory protein 1 alpha; GzB, granzyme B; GM-CSF, granulocyte macrophage colony-stimulating factor; MCP-1, monocyte chemoattractant protein-1; MIP-1β, macrophage inflammatory protein-1β; TNFRp55, tumor necrosis factor receptor p55; Ang, angiopoietin; TNF-α, tumor necrosis factor alpha.

#### ICANS grading standards

Before the development of the ASTCT consensus criteria, the CTCAE and CARTOX criteria were used to grade CAR-T cell-related neurotoxicity (Table 7). The ASTCT consensus guidelines were modified based on the CARTOX criteria, and the CARTOX-10 score was slightly modified to the immune effector cell-associated encephalopathy (ICE) score (Table 8), forming the ASTCT neurotoxicity (called ICANS) grading standard (Lee et al., 2019). To assess the mental status after CAR-T cell therapy, the ASTCT consensus group recommends the ICE score for adults and the Cornell Assessment of Pediatric Delirium (CAPD) for children (<12 years old) (Traube et al., 2014; Silver et al., 2015; Maus et al., 2020). The ASCO guidelines published in 2021 refer to the ASTCT consensus to classify ICANS and formulate management strategies for ICANS based on a multidisciplinary approach and relevant published evidence (Santomasso et al., 2021).

**TABLE 7:** Grading of ICANS.

ICANS grading system	CTCAEv5.0	CARTOX criteria (Neelapu et al., 2018a)	ASTCT consensus criteria (Lee et al., 2019)	ASCO guideline (Santomasso et al., 2021)
Grade 1	• Encephalopathy: mild symptoms	• CARTOX-10 score 7–9 (mild impairment)	• ICE score 7–9	• ICE score 7–9 with no depressed level of consciousness
	• Seizure: brief partial seizure and no loss of consciousness	• No raised intracranial pressure	• CAPD score 1–8	
	• Dysphasia: awareness of receptive or expressive characteristics; Not impairing ability to communicate	• No seizures or motor weakness	• And/or depressed level of consciousness but awakens spontaneously	
	• Tremor: mild symptoms		• No seizures	
	• Headache: mild pain		• No motor weakness	
	• Confusion: mild disorientation		• No elevated ICP/cerebral edema	
	• Depressed level of consciousness: decreased level of alertness			
Grade 2	• Encephalopathy: moderate symptoms; Limiting instrumental ADL	• CARTOX-10 score 3–6 (moderate impairment)	• ICE score 3–6	• ICE score 3–6
	• Seizure: brief generalized seizure	• No raised intracranial pressure	• CAPD score 1–8	• And/or Mild somnolence awaking to voice
	• Dysphasia: moderate receptive or expressive characteristics; Impairing ability to communicate spontaneously	• No seizures or motor weakness	•And/or depressed level of consciousness but awakens to voice	
	• Tremor: moderate symptoms; Limiting instrumental ADL		• No seizures	
	• Headache: moderate pain; Limiting instrumental ADL		• No motor weakness	
	• Confusion: moderate disorientation; Limiting instrumental ADL		• No elevated ICP/cerebral edema	
	• Depressed level of consciousness: sedation; Slow response to stimuli; Limiting instrumental ADL			
Grade 3	• Encephalopathy: severe symptoms; Limiting self-care ADL	• CARTOX-10 score 0–2 (severe impairment)	• ICE score 0–2	• ICE score 0–2
	• Seizure: new-onset seizures (partial or generalized); Multiple seizures despite medical intervention	• Stage 1–2 papilloedema, or CSF opening pressure <20 mmHg	• CAPD score ≥9	• And/or depressed level of consciousness awakening only to tactile stimulus
	• Dysphasia: severe receptive or expressive characteristics; Impairing ability to read, write, communicate intelligibly	• Partial seizure, or non-convulsive seizures on EEG with response to benzodiazepine	• And/or depressed level of consciousness but awakens to tactile stimulus	• And/or any clinical seizure focal or generalized that resolves rapidly or nonconvulsive seizures on EEG that resolve with intervention
	• Tremor: severe symptoms; limiting self-care ADL		• Any clinical seizure focal or generalized that resolves rapidly or nonconvulsive seizures on EEG that resolve with intervention	• And/or focal or local edema on neuroimaging
	• Headache: severe pain; Limiting self-care ADL		• No motor weakness	
	• Confusion: severe disorientation; Limiting self-care ADL		• Focal/local edema on neuroimaging	
	• Depressed level of consciousness: difficult to arouse			
	• Cerebral edema: new onset; Worsening from baseline			
Grade 4	Life-threatening consequences; urgent intervention indicated	• Unable to perform CARTOX-10	• ICE score 0 (unable to perform ICE)	• ICE score 0 (unable to perform ICE)
		•Stage 3–5 papilloedema, or CSF opening pressure ≥20 mmHg, or cerebral oedema	• unable to perform CAPD	•And/or stupor or coma
		•Generalized seizures, or convulsive or non-convulsive status epilepticus, or new motor weakness	• patient is unarousable or requires vigorous or repetitive tactile stimuli to arouse (stupor or coma)	•And/or life-threatening prolonged seizure (≻ 5 min) or repetitive clinical or electrical seizures without return to baseline in between •And/or diffuse cerebral edema on neuroimaging, decerebrate or decorticate posturing or papilledema, cranial nerve VI palsy, or Cushing’s triad
			• Life-threatening prolonged seizure (>5 min); Or repetitive clinical or electrical seizures without return to baseline in between • Deep focal motor weakness such as hemiparesis or paraparesis	
			• Diffuse cerebral edema on neuroimaging; decerebrate or decorticate posturing; or cranial nerve VI palsy; or papilledema; or Cushing’s triad	
Grade 5	Death	—	Death due to ICANS	—

Papilloedema grading is performed according to the modified Frisén scale (Frisen, 1982).

ICANS, immune effector cell-associated neurotoxicity syndrome; CTCAE, Common Terminology Criteria for Adverse Events; ADL, indicates activities of daily living; CARTOX, CAR-T cell therapy associated toxicity; CARTOX-10, CAR-T cell therapy associated toxicity 10-point neurological assessment; CSF, cerebrospinal fluid; EEG, electroencephalogram; ASTCT, American Society for Transplantation and Cellular Therapy; ICE, Immune Effector Cell-Associated Encephalopathy score; CAPD, Cornell Assessment of Pediatric Delirium; ICP, intracranial pressure; ASCO, American Society of Clinical Oncology.

**TABLE 8:** Encephalopathy assessment tools for grading of ICANS.

CARTOX-10 (Neelapu et al., 2018a)	ICE (Lee et al., 2019)
• Orientation (5 points): orientation to year, month, city, hospital, president/prime minister of country of residence	• Orientation (4 points): orientation to year, month, city, hospital
• Naming (3 points): name three objects (e.g., point to clock, pen, button)	• Naming (3 points): name three objects (e.g., point to clock, pen, button)
• Writing (1 points): write a standard sentence (e.g., “Our national bird is the bald eagle”)	• Writing (1 points): write a standard sentence (e.g., “Our national bird is the bald eagle”)
• Attention (1 points): count backwards from 100 in 10	• Attention (1 points): ability to count backwards from 100 by 10
	• Following commands (1 points): follow simple commands (e.g., “Show me 2 fingers” or “Close your eyes and stick out your tongue”)
Grade 1 ICANS: 7–9 points	
Grade 2 ICANS: 3–6 points	
Grade 3 ICANS: 0–2 points	
Grade 4 ICANS: unarousable, unable to complete assessment	

CARTOX-10, CAR-T cell therapy associated toxicity 10-point neurological assessment; ICE, immune Effector Cell-Associated Encephalopathy score; ICANS, immune effector cell-associated neurotoxicity syndrome.

#### Therapeutic measures for ICANS

One study found that the prophylactic use of tocilizumab reduced the incidence of severe CRS and did not increase the risk of ICANS when infused with anti-CD19 CAR-T cells containing CD3ζ/4-1BB costimulatory signaling to treat NHL patients (Caimi et al., 2021). However, some studies reveal that the prophylactic use of tocilizumab increases the incidence of severe ICANS (Totzeck et al., 2022). Therefore, the prophylactic use of tocilizumab requires more evaluations and trials for verification. Ragoonanan et al. (2021) revealed that if ICANS and CRS coexist, tocilizumab is recommended for any grade of ICANS, and dexamethasone or methylprednisolone can be given if tocilizumab is ineffective. However, Norelli et al. (2018) found that blocking IL-6 receptors with tocilizumab could treat CRS in mouse models. However, this was ineffective against delayed fatal neurotoxicity. Santomasso et al. (2018) also reported that administering tocilizumab was ineffective in most patients with neurotoxicity. A possible reason is that tocilizumab does not easily cross the blood-brain barrier, and its administration leads to a compensatory increase in IL-6 in the CNS, which eventually aggravates ICANS (Nishimoto et al., 2008; Nellan et al., 2018). Therefore, systemic corticosteroid dexamethasone is recommended as a first-line treatment for grade 2–3 ICANS, and dexamethasone (10 mg, IV) is recommended every 6–8 h (Brudno and Kochenderfer, 2016; Neelapu, 2019; Schubert et al., 2021). High-dose methylprednisolone (1000 mg, IV) is recommended for 3 days when dexamethasone is ineffective or if ICANS is grade 4 (Gust et al., 2017; Schubert et al., 2021). Studies have shown that the prompt use of corticosteroids prevents severe ICANS without influencing the efficacy of CAR-T cell therapy (Topp et al., 2020). Holtzman et al. (2021) also confirmed that corticosteroids could be used to treat ICANS without compromising CAR-T efficacy. However, they mentioned that the rapid reduction in corticosteroids could trigger the onset of ICANS. Therefore, high doses of corticosteroids should be slowly reduced and closely monitored for recurrence of ICANS. The optimal dose and duration of corticosteroid administration remain uncertain, and further research is needed to determine whether long-term high-dose corticosteroids affect the therapeutic effect of CAR-T therapy.

Unlike tocilizumab, siltuximab binds to circulating IL-6 and further reduces active IL-6 in the CNS; therefore, it could be an effective drug for treating patients who did not respond to tocilizumab or corticosteroids (Riegler et al., 2019). However, further clinical trials are needed to confirm this finding. In addition, the IL-1 receptor antagonist, anakinra, showed an excellent therapeutic effect on CRS and ICANS and can be an effective drug for treating steroid-refractory ICANS with or without CRS (Norelli et al., 2018; Wehrli et al., 2022). Relevant clinical trials on the early prophylactic use of anakinra are also underway (Table 5). Sachdeva et al. (2019) found that when CAR-T cells were knocked out for GM-CSF, CRS-related inflammatory cytokines released by monocytes decreased. In another *in vivo* study, Sterner et al. (2019) found that blocking GM-CSF improved CRS and ICANS while enhancing the antitumor activity of CAR-T cells in mouse models. Blocking GM-CSF is a possible mechanism for treating CRS; however, more trials are needed to support its application in humans. For high-risk patients with ICANS, levetiracetam 750 mg orally or intravenously every 12 h on the day of CAR-T cell infusion is recommended to prevent seizures (Neelapu et al., 2018a). Antiepileptic drugs, such as levetiracetam, phenobarbital and benzodiazepines, are known for treating epilepsy (Neelapu, 2019). In conclusion, no substantial evidence exists that blocking a single cytokine can prevent or improve ICANS. Moreover, since the pathogenesis of ICANS is still unclear, the treatment of ICANS is mostly symptomatic and not causative.

According to the management opinions (Table 9) put forward by each guideline, management is mainly performed for ICANS without concurrent CRS and ICANS with concurrent CRS. Tocilizumab (8 mg/kg, IV) is recommended for treating ICANS in patients with concurrent CRS. Dexamethasone (10 mg q6h, IV) and methylprednisolone (1 mg/kg q12h, IV) were administered to treat ICANS without concurrent CRS or ICANS with concurrent CRS that did not respond to anti-IL-6 therapy. For life-threatening grade 4 ICANS, high-dose methylprednisolone (1 g, IV) could be used as maintenance therapy until it improves to grade 1 and then slowly tapered (Table 9). The therapeutic dose of dexamethasone and time interval for different grades of ICANS were slightly different (Table 9). In addition to the administration of drugs, daily supportive care and neurological examinations should be performed for patients with ICANS.

**TABLE 9:** Management of ICANS.

ICANS management system	CARTOX criteria (Neelapu et al., 2018a)	ASTCT consensus criteria (Neelapu, 2019; Castaneda-Puglianini and Chavez, 2021)	ASCO guideline (Santomasso et al., 2021)
Grade 1	• Supportive care, aspiration precautions, IV hydration	• Aspiration precautions and IV hydration	• No concurrent CRS: offer supportive care with IV hydration and aspiration precautions
	• Low doses of lorazepam (0.25–0.5 mg q8h, IV) or haloperidol (0.5 mg q6h, IV) can be used, for agitated patients	• Seizure prophylaxis with levetiracetam	• With concurrent CRS: administer tocilizumab (8 mg/kg, IV); Repeat q8h as needed; Limit to a maximum of three doses in a 24 h period; Maximum total of four doses; Caution with repeated tocilizumab doses in patients with ICANS; Consider adding corticosteroids to tocilizumab past the first dose
	• MRI of the brain with and without contrast, CT scan of the brain can be performed if MRI of the brain is not feasible	• EEG	
	• Daily 30 min EEG until toxicity symptoms resolve	• Imaging of brain (MRI preferred if no contraindication)	
	• Levetiracetam (750 mg, q12h) to prevent epilepsy	• Consider tocilizumab if there is concurrent CRS	
	• Tocilizumab (8 mg/kg, IV) or siltuximab (11 mg/kg, IV), if ICANS is associated with concurrent CRS	• Neurocognitive assessment q6h using ICE scoring system	
Grade 2	• Supportive care and neurological work-up as described for grade 1 ICANS	• Supportive care as in grade 1	• No concurrent CRS: offer supportive care as per grade 1; For high-risk products or patients, consider dexamethasone (10 mg, IV) two doses (or equivalent) and reassess. Repeat q 6–12 h if no improvement; Taper steroids as clinically appropriate once symptoms improve to grade 1
	• Tocilizumab (8 mg/kg, IV) or siltuximab (11 mg/kg, IV) if associated with concurrent CRS	• Consider dexamethasone (10 mg q6h, IV) or its equivalent of methylprednisolone	• With concurrent CRS: consider ICU transfer if ICANS associated with ≥ grade 2 CRS; Administer tocilizumab as per grade 1; If refractory to tocilizumab past the first dose, initiate dexamethasone (10 mg q6-12h, IV) or methylprednisolone equivalent (1 mg/kg q12h, IV) until improvement to grade 1, and then taper
	• Dexamethasone (10 mg q6h, IV) or methylprednisolone (1 mg/kg q12h, IV) if refractory to anti-IL-6 therapy or for ICANS without concurrent CRS	• Tocilizumab if concurrent CRS	
	• Consider transferring patient to ICU if ICANS associated with grade ≥2 CRS		
Grade 3	• Supportive care and neurological work-up as described for grade 1 ICANS	• Supportive care as in grade 1	• Transfer patient to ICU
	• Tocilizumab (8 mg/kg, IV) or siltuximab (11 mg/kg, IV) if associated with concurrent CRS	• Dexamethasone (10–20 mg q6h, IV) or its equivalent of methylprednisolone	• No concurrent CRS: administer dexamethasone (10 mg q6h, IV) or methylprednisolone equivalent (1 mg/kg q12h, IV)
	• Corticosteroids as outlined for grade 2 ICANS if symptoms worsen despite anti-IL-6 therapy, or for ICANS without concurrent CRS	• Control seizures with benzodiazepines (for short-term control) and levetiracetam ± phenobarbital and/or lacosamide	• With concurrent CRS: administer tocilizumab as per grade 1; If refractory to tocilizumab past the first dose, initiate dexamethasone (10 mg q6h, IV) or methylprednisolone equivalent (1 mg/kg q12h, IV) until improvement to grade 1, and then taper
	• ICU transfer and repeat neuroimaging (CT or MRI every 2–3 days) are recommended	• High-dose methylprednisolone (1 g/day) for focal/local edema	
		• Transfer to ICU	
		
Grade 4	• Supportive care and neurological work-up as described for grade 1 ICANS	• Supportive care as in grade 1	• Admit patient to ICU
	•Anti-IL-6 therapy and repeat neuroimaging as described for grade 3 ICANS	• High-dose methylprednisolone (1 g/day) for 3 days followed by taper	• No concurrent CRS: administer high-dose methylprednisolone (1g, IV) one to two times per day for 3 days; If not improving, consider 1g of methylprednisolone two to three times per day or alternate therapy; Continue corticosteroids until improvement to grade 1, and then taper; Status epilepticus to be treated as per institutional guidelines
	• High-dose corticosteroids methylprednisolone (1 g/day, IV) for 3 days continued until improvement to grade 1 ICANS and then taper	• Control seizures with benzodiazepines (for short-term control) and levetiracetam ± phenobarbital and/or lacosamide	
	•ICU monitoring, consider mechanical ventilation for airway protection	• Imaging of spine for focal motor weakness	
		• Lower ICP by hyperventilation, hyperosmolar therapy with mannitol/hypertonic saline, and/or neurosurgery consultation for ventriculoperitoneal shunt in patients with cerebral edema	• With concurrent CRS: administer tocilizumab as per grade 1 in addition to methylprednisolone (1g, IV) one to two times per day for 3 days; If not improving, consider methylprednisolone (1g, IV) two to three times a day or alternate therapy; Continue corticosteroids until improvement to grade 1, and then taper
		• Transfer to ICU	

Tocilizumab IV over 1 h, Maximum amount of tocilizumab per dose is 800 mg.

ICANS, immune effector cell-associated neurotoxicity syndrome; CARTOX, CAR-T cell therapy associated toxicity; IV, intravenous; q6h, every 6 hours; q8h, every 8 hours; q12h, every 12 hours; MRI, magnetic resonance imaging; CT, computed tomography; EEG, electroencephalogram; CRS, cytokine release syndrome; ICU, intensive-care unit; ASTCT, American Society for Transplantation and Cellular Therapy; ICE, immune Effector Cell-Associated Encephalopathy score; ICP, intracranial pressure; ASCO, American Society of Clinical Oncology.

## Organ system toxicities of CAR-T cell therapy

### Cardiovascular toxicity concurrent cytokine release syndrome

CRS is also a crucial factor that induces adverse cardiovascular events that can lead to severe cardiovascular complications. Similar to other systemic inflammatory response syndromes, sinus tachycardia and hypotension are the most common clinical signs (Totzeck et al., 2022). Fever caused by CRS is the inducement of sinus tachycardia (Ghosh et al., 2020). Other cardiovascular complications associated with CRS include increased serum troponin levels, decreased left ventricular ejection fraction (LVEF), cardiogenic shock, arrhythmias, corrected QT prolongation, decompensated heart failure, and cardiovascular death (Asnani, 2018; Burstein et al., 2018; Ganatra et al., 2019a; Alvi et al., 2019). Cardiotoxicity occurs between 2 and 24 days after CAR-T cell infusion (Ganatra et al., 2020; Lefebvre et al., 2020). Alvi et al. (2019) found in a retrospective cohort study of 137 patients treated with CAR-T that cardiovascular events occurred only in cases of CRS ≥ grade 2, with an incidence of 12–28%. Ganatra et al. (2020) performed echocardiographic follow-up in 116 patients with CRS ≥ grade 2 and found that 10.3% developed new or worsening cardiomyopathy. Elevated troponin and inappropriate administration of tocilizumab in CRS patients after CAR-T infusion are associated with an increased risk of subsequent cardiovascular events (Alvi et al., 2019; Ghosh et al., 2020).

Children with hematological cancers have a higher incidence of adverse cardiovascular events after CAR-T therapy. Fitzgerald et al. (2017) analyzed 39 pediatric patients and found that 14 (36%) developed cardiovascular dysfunction after CAR-T therapy. Another study of 98 pediatric patients revealed hypotension in 24 patients (24%) and life-threatening hypotension in 21 patients (21%) (Burstein et al., 2018). In an adult CAR-T-related cardiovascular adverse event study, Alvi et al. (2019) found that 17 (12%) patients experienced cardiovascular events such as cardiovascular death, decompensated heart failure, and arrhythmias. In another study, Lefebvre et al. (2020) found that 31 adult patients (21%) developed major adverse cardiovascular events (MACE), including heart failure and arrhythmia. Although CRS is the leading cause of cardiovascular toxicity during CAR-T therapy, other factors, such as tumor lysis syndrome (TLS), infection, and primary cardiovascular events, need to be excluded. In addition to treating hematological cancers, CAR-T targeting fibroblast activating protein (FAP) is effective in mice with cardiac fibrosis (Aghajanian et al., 2019). CAR-T cell therapy exhibits cardiovascular toxicity and the potential to treat heart diseases.

#### Pathogenesis of cardiovascular toxicity

**FIGURE 3:**
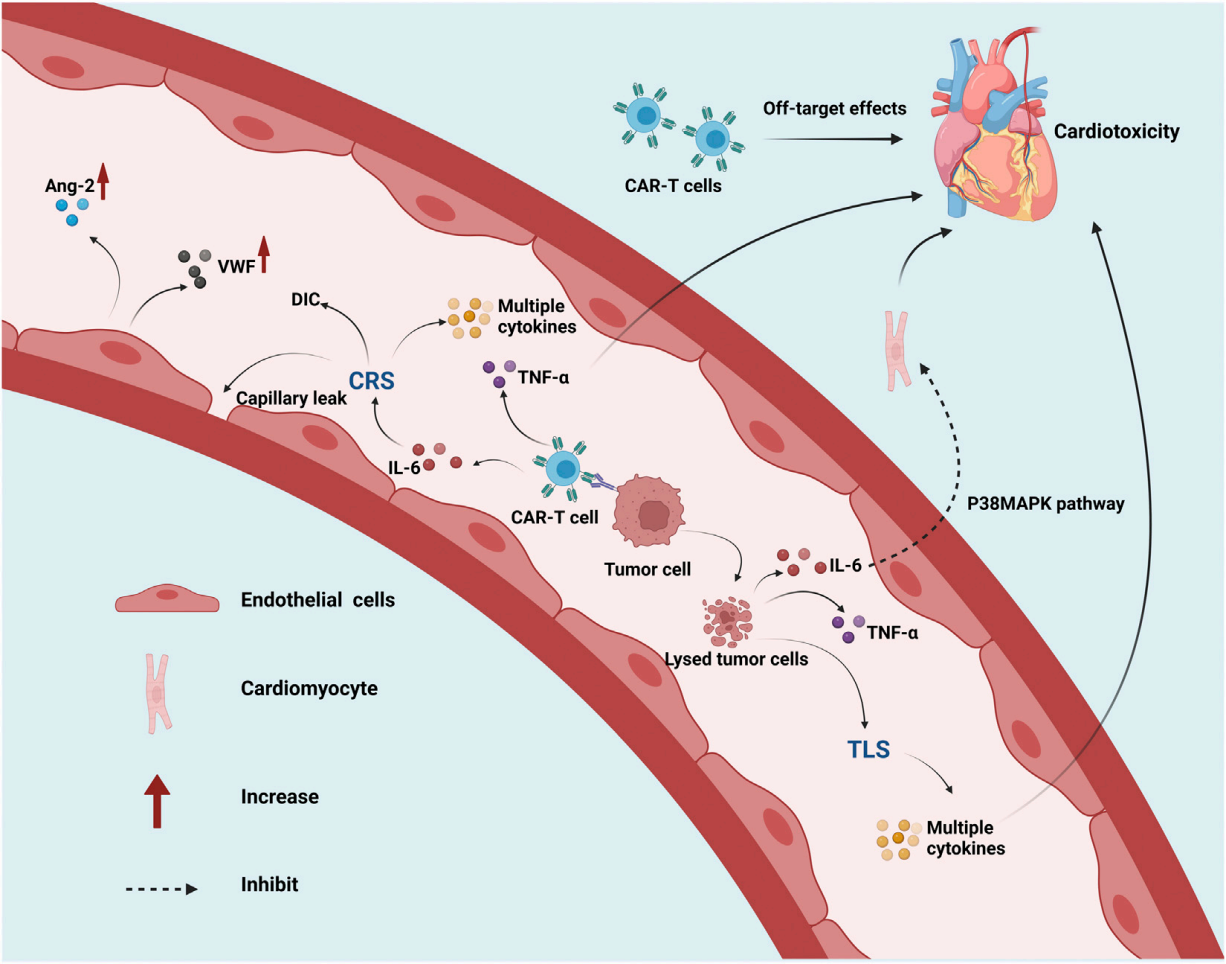
Pathogenesis of cardiovascular toxicity during CAR-T therapy. Abbreviations: CAR, chimeric antigen receptor; CRS, cytokine release syndrome; IL, interleukin; TNF-α, tumor necrosis factor alpha; MAPK, mitogen-activated protein kinase; TLS, tumor lysis syndrome; DIC, disseminated intravascular coagulation; Ang-2, angiopoietin-2; VWF, von willebrand factor. This figure created with BioRender.com.

The specific mechanism of cardiovascular adverse reactions in CAR-T treatment is not precise, and the potential mechanisms (Figure 3) include: *1*) severe CRS results in hemodynamic instability, capillary leakage, and DIC, and increased serum concentrations of VWF and Ang-2 (Hay et al., 2017); *2*) IL-6 is a crucial cytokine leading to CAR-T therapy-related CRS, and a significant increase in IL-6 is closely related to adverse cardiovascular reactions (Stein-Merlob et al., 2021). Pathan et al. (2011) found that IL-6 (serum endothelial activating cytokine) inhibits the contractile function of myocardium through the p38MAPK signaling pathway; *3*) Increased expression of TNF-α in the myocardium enhances cardiotoxicity (Michel et al., 2022); *4*) Direct cardiotoxicity caused by off-target cross-reaction of CAR-T cells to actin (Linette et al., 2013); *5*) Arrhythmias induced by TLS-related metabolic disorders (Ganatra et al., 2019b).

#### Risk factors of cardiovascular toxicity

Currently, there is no exact cardiovascular risk assessment method, and formulating relevant rules requires multidisciplinary cooperation. High-risk factors or predictors of severe cardiovascular toxicity include *1*) cardiotoxic therapy such as anthracyclines and chest radiotherapy (Ghosh et al., 2020); *2*) Patients with cardiovascular complications such as hypertension, atrial fibrillation/flutter, coronary ischemia, and structural heart disease (Ganatra et al., 2019a); *3*) Higher age, CRS grade ≥2 and hyperlipidemia are all risk factors for inducing cardiovascular toxicity in CAR-T cell therapy (Alvi et al., 2019; Ganatra et al., 2020); *4*) Higher baseline creatinine levels were independently associated with MACE, and the use of statins, insulin, and aspirin was associated with adverse cardiovascular reactions (Lefebvre et al., 2020).

#### Monitoring and treatment of cardiovascular toxicity

Although cardiovascular adverse events may be transient and reversible in patients with sufficient cardiovascular reserve, they are particularly challenging for high-risk patients (Ganatra et al., 2019a). It is important to identify and predict patients at risk of fatal cardiotoxicity is crucial for initiating early interventions and reducing the risk of CAR-T therapy. Exercise tolerance should be evaluated in patients with a history of cardiovascular disease and cardiovascular abnormalities detected in the initial examination, and further tests should be performed to rule out potential occult coronary ischemia or other structural heart diseases to assess tolerance to hemodynamic changes induced by CRS after CAR-T therapy (Ganatra et al., 2019a). Shalabi et al. (2020) proposed early intervention for possible severe vascular toxicity through monitoring and analysis of echocardiography, baseline LV global longitudinal strand (GLS), and cardiac biomarkers (troponin and pro-B-type native peptide). In addition, Totzeck et al. (2022) suggested that electrocardiogram (ECG), echocardiography, high-sensitivity cardiac troponin (hscTn), N-terminal pro-brain natriuretic peptide (NT-proBNP), and other examinations should be performed on the seventh day of CAR-T treatment. High-risk patients should be followed up for 3 months, to determine early and late cardiovascular toxicity. Cardiovascular assessment before and during CAR-T therapy is helpful for the early identification of patients with insufficient cardiovascular reserve. Before treatment, the disease status of the patient, medication, and treatment histories should be investigated in detail. In addition, the cardiac function of the patient should be monitored using 12-lead ECG, echocardiography, cardiac biomarkers and other methods.

First, patients with existing cardiovascular disease should be actively treated with drugs to control the disease. Second, the occurrence of CRS and cardiovascular toxicity in the early stage of CAR-T immunotherapy should be assessed for severity (Totzeck et al., 2022). Tocilizumab should be a priority when CRS is combined with cardiotoxicity, followed by glucocorticoids if the condition cannot be controlled (Schuster et al., 2017). Abnormal levels of cardiac biomarkers before and after CAR-T therapy and left ventricular systolic dysfunction (LVSD) are found on transthoracic echocardiography (TTE) or cardiac magnetic resonance (CMR) imaging, prompting β-blockers, angiotensin-converting enzyme inhibitors, or angiotensin receptor blockers to be used as cardiac protection (Ghosh et al., 2020). A retrospective study found that 94% of patients with elevated troponin and CRS ≥2 grade had cardiovascular toxicity, and elevated troponin levels are a possible indication for treatment with tocilizumab (Alvi et al., 2019). The results showed that patients could benefit from early tocilizumab treatment when troponin levels are elevated (Alvi et al., 2019).

### Hematologic and infectious toxicity

CAR-T cell therapy-related hematologic toxicities include cytopenia (neutropenia, thrombocytopenia, leucopenia, anemia, or any combination of these), B-cell aplasia, and coagulation disorders. Among them, the incidence of neutropenia is 33–94%, thrombocytopenia is 30–80%, leucopenia is 31–47%, and anemia is 30–68% (Zhao et al., 2018; Fried et al., 2019; Locke et al., 2019; Schuster et al., 2019). Lymphodepleting chemotherapy is considered the leading cause of early cytopenia, but the cause of late cytopenias remains unclear (Fried et al., 2019). Close attention to possible herpes zoster and *Pneumocystis jirovecii* pneumonia and drug prophylaxis for at least 1 year is recommended if fludarabine is used for lymphodepletion chemotherapy (Neelapu, 2019). Persistent cytopenia is associated with infectious complications such as late fatal encephalitis and systemic mycosis (Kansagra et al., 2019). CAR-T cell infusion is not recommended for patients with bacterial infection if the fever is not well controlled and the bacterial culture is not negative for 48 h (Maus et al., 2020). Furthermore, patients whose viral and fungal infections have not been effectively controlled are not recommended to continue CAR-T therapy (Maus et al., 2020). Prophylaxis against infections with antibacterial and fungal agents should be considered in patients with prolonged grade 4 neutropenia (Neelapu, 2019). Fried et al. (2019) showed that patients with severe CRS and recent stem cell transplantation (<1 year) were more likely to develop late hematologic toxicity and that serum SDF-1 levels were associated with neutropenia. In the ASCO guidelines, granulocyte-colony stimulating factor (G-CSF) rather than GM-CSF is considered to treat CAR-T-induced neutropenia (Santomasso et al., 2021). Furthermore, G-CSF is strongly recommended for the treatment of long-term neutropenia; however, to avoid interaction with the peak CRS risk and CAR-T expansion period, only use G-CSF after 14 days of CAR-T cell infusion or CRS resolution (Maus et al., 2020; Schubert et al., 2021). GM-CSF is not recommended because it can aggravate CRS (Maus et al., 2020). Patients with thrombocytopenia are at an increased risk of gastrointestinal, genitourinary, intracranial, and pulmonary bleeding and should be closely monitored for 1 month after CAR-T therapy (Johnsrud et al., 2021).

CD19 is expressed in normal B-cells and B-cell malignancies; therefore, B-cell aplasia is a common toxicity of anti-CD19 CAR-T therapy (Townsend et al., 2018; Roddie et al., 2021). The significant signs were low B-cell counts and immunoglobulin levels (Santomasso et al., 2021). Fortunately, B-cell aplasia is clinically tolerated because hypogammaglobulinemia resulting from CD19 ablation of B cells can be managed with intravenous immunoglobulin (Maude et al., 2018; Miao et al., 2021). Approximately 51–56.6% of patients with hematologic malignancies develop coagulopathy after CAR-T cell therapy (Miao et al., 2021). Further deterioration of coagulopathy can cause DIC, and patients with severe CRS have a higher incidence of coagulopathy and DIC (Miao et al., 2021).

### HLH/MAS

In severe CRS, ferritin is considerably elevated, accompanied by high fever, hepatosplenomegaly, hemocytopenia, and coagulopathy, revealing the possible occurrence of hemophagocytic lymphohistiocytosis (HLH)/macrophage activation syndrome (MAS) (Pehlivan et al., 2018; Frigault and Maus, 2020; Spiegel et al., 2021). The incidence of HLH/MAS in CAR-T cell therapy was approximately 3.48%, but the mortality rate was up to 80%, and the prognosis was poor (Alblooshi et al., 2020; Sandler et al., 2020a; Sandler et al., 2020b).

HLH/MAS should be considered in CRS patients with peak serum ferritin levels >10,000 ng/ml within 5 days after CAR-T infusion and any two of the following: grade ≥3 organ toxicity involving the liver, kidney, or lung (according to CTCAEv5.0), or hemophagocytosis in the bone marrow or other organs (Neelapu et al., 2018a). In patients with suspected secondary HLH/MAS, testing for fasting triglycerides and soluble IL-2R is recommended (Ragoonanan et al., 2021). Currently, no targeted therapies are available for patients with HLH/MAS. In principle, more aggressive immunosuppressive therapy should be administered at an early stage, with glucocorticoids and tocilizumab as the mainstay of treatment (Neelapu et al., 2018a; Miao et al., 2021). Etoposide should only be used in patients with late-onset HLH/MAS who are refractory to tocilizumab (Maus et al., 2020). Moreover, for the treatment of late-onset HLH/MAS, third-line CRS agents such as anakinra at starting doses of 5–8 mg/kg/day should be considered (Maus et al., 2020; Shah et al., 2020). Other treatments for late-onset HLH/MAS include intrathecal methotrexate and cytarabine, but these are controversial and lack formal assessment (Horne et al., 2017; Neelapu et al., 2018b; Hashmi et al., 2019).

### Skin toxicity

The incidence of rash in FDA-approved anti-CD19 CAR-T therapies (axicabtagene ciloleucel, tisagenlecleucel, and brexucabtagene autoleucel) is 9–22% (Nusbaum et al., 2021). However, attention should be paid to distinguishing an allergic skin reaction caused by chemotherapy, antibacterial agents, DMSO, or other drugs. CAR-T skin toxicities usually manifest as papules, maculopapular eruptions, purpura, urticarial rash, bullous eruptions, dry skin, and oral mucositis (Rubin et al., 2016; Wang et al., 2017; Hu et al., 2020; Ramos et al., 2020; Nusbaum et al., 2021). Skin toxicity occurs 5 days to 19 months after CAR-T infusion (Rubin et al., 2016; Hu et al., 2020). However, there are few reports on cases, literature reviews, and clinical trials of skin toxicity caused by CAR-T cell therapy; thereby providing a gap for research.

Wang et al. (2017) found that 11.1% of patients developed urticarial-like rashes after infusion of anti-CD30 CAR-T cells. Another study elaborated that 48% of patients developed maculopapular rashes after infusion of anti-CD30 CAR-T cells; these rashes were transient and did not require specific treatment (Ramos et al., 2020). Rubin et al. (2016) reported adverse skin reactions, including secondary cutaneous malignancies, disseminated infection, eruptions with unusual mononuclear cell dermal infiltrate, and transient eruptions suggestive of the “eruption of lymphocyte recovery” after anti-CD19 CAR-T treatment in five patients. In phase I clinical trials of anti-EGFR CAR-T cells in the treatment of metastatic pancreatic cancer, dry skin, dermatitis herpetiformis, oral mucositis, and other skin toxicities were found (Liu et al., 2020). In addition, one case reported that the patient developed a diffuse maculopapular rash 5 days after CAR-T infusion, which then evolved into tension bullae (Hu et al., 2020). The main cells in the bullous fluid are CAR-T cells, and the concentrations of IL-6 and IFN-γ in the bullous fluid are significantly higher than those in serum (Hu et al., 2020). IL-6 is a critical cytokine in CRS, and reduced immune function induced by CRS lead to skin infections in such patients. Moreover, the secretion of other pro-inflammatory cytokines during CAR-T therapy exacerbates the severity of skin reactions. Skin toxicity can cause psychological and physical harm to patients, and severe skin toxicity can lead to death. As CAR-T therapy becomes more widely used in cancer treatment, doctors should pay attention to the possible adverse skin reactions and manage patients accordingly.

### Other organ toxicities concurrent cytokine release syndrome

CRS is associated with various clinical findings, including fever and multiple organ dysfunction (pulmonary, renal, hepatotoxicity, and gastrointestinal toxicity) (Ghosh et al., 2020). In a pharmacovigilance and meta-analysis study, it was found that the incidence of CAR-T cell-related pulmonary toxicity (respiratory failure) was 9.0%, nephrotoxicity (acidosis, adrenal insufficiency, electrolyte disturbances, and renal failure) was 6.0%, and hepatotoxicity (liver injury) was 1.5% (Dolladille et al., 2021). The results of phase I/II clinical studies by Hay et al. (2017) showed that 3 (30.0%) patients with grade ≥4 CRS developed grade ≥3 acute kidney injury, one patient required hemodialysis for 15 days, and nine patients developed liver dysfunction (elevated aspartate aminotransferase, alanine aminotransferase, alkaline phosphatase, and bilirubin). In addition to causing pulmonary, hepatic, and renal toxicity, gastrointestinal toxicity also occurred after the infusion of CAR-T cells. The most common gastrointestinal toxicities were diarrhea, vomiting, bleeding, and nausea. (Zeng et al., 2020). Furthermore, Zeng et al. (2020) showed that damage to the mucosal barrier leads to the spread of bacteria from the gastrointestinal tract into the blood, resulting in bacteremia and sepsis if patients present with simultaneous gastrointestinal bleeding and diarrhea. Severe CRS can easily lead to severe multi-organ system toxicity, which is one of the main reasons limiting the safe application of CAR-T therapy.

## Potential cytokine release syndrome therapeutic drugs and methods

Tocilizumab is an FDA-approved treatment of CAR-T treatment-induced CRS. Corticosteroids are also used in the treatment of CRS as first- and second-line therapies. As a tocilizumab congener, siltuximab blocks IL-6 signaling by binding to IL-6. As siltuximab has a higher affinity for IL-6 than tocilizumab for IL-6R, it is considered another potential drug for the treatment of CRS. If the patient does not respond to tocilizumab and corticosteroids, siltuximab may be administered at a dose of 11 mg/kg BW/dose (Riegler et al., 2019; Schubert et al., 2021). However, the FDA has not approved siltuximab for the treatment of CAR-T cell-induced CRS and ICANS. In addition to anti-IL-6 therapies, ongoing trials are exploring the use of the JAK1 selective inhibitor itacitinib to prevent CRS induced by tisagenlecleucel or axicabtagene ciloleucel (NCT04071366) and the JAK 1/2 inhibitor ruxolitinib for the treatment of HLH/MAS (Ahmed et al., 2019; Ragoonanan et al., 2021). In mouse models of CRS, studies have shown that administering a short course of the tyrosine kinase inhibitor dasatinib early after CAR-T cell infusion can avoid fatal CRS by inhibiting CAR-T cell functions, such as proliferation and cytokine secretion (Mestermann et al., 2019; Weber et al., 2019). In addition, ibrutinib was found to attenuate CRS while increasing antitumor efficacy in mice treated with CAR-T (Fraietta et al., 2016; Ruella et al., 2017). Patients who received ibrutinib 2 weeks before surgery and 3 months after CAR-T cell infusion had lower CRS severity than those who did not receive ibrutinib (Sheth and Gauthier, 2021). In addition to the above drugs, TNF-α inhibitors, etanercept, and infliximab; IL-1 inhibitor, anakinra; IFN-γ inhibitor fontolizumab, and others are promising drugs for the treatment of CRS (Riegler et al., 2019).

Similar to the treatment of uremia, removing harmful substances and pro-inflammatory cytokines from blood is an effective treatment for severe CRS that is ineffective with current drug therapy. A 23-year-old man with a typical CRS response was effectively controlled with dexamethasone (10 mg, q6h) and plasmapheresis after failing to control his condition with glucocorticoids and tocilizumab (Xiao et al., 2019). Another study showed that when tocilizumab and glucocorticoid therapy were ineffective in controlling CAR-T cell-induced adverse effects, hemofiltration immediately ameliorated severe CRS and induced multiple organ dysfunction (Liu et al., 2018). In addition, the treatment of a 65-year-old man with grade 4 CRS with *in vitro* cytokine adsorption showed a more than 50% reduction in multiple pro-inflammatory cytokines levels (Stahl et al., 2020).

Another way to reduce the adverse reactions of CAR-T cells from the root and improve their safety is to set a “suicide” switch on CAR-T cells. When CAR-T cells are infused, this switch activates and consumes CAR-T cells on demand at the desired time. Commonly used “suicide switches” include inducible caspase 9 (iC9), herpes simplex virus thymidine kinase (HSV-TK), CD20, and truncated epidermal growth factor receptor (EGFRt) (Gargett and Brown, 2014; Greco et al., 2015; Yu et al., 2019; Klopp et al., 2021; Warda et al., 2021). Among these, HSV-TK and iC9 have been integrated into CAR-T cells and tested clinically (Andrea et al., 2020). Because HSV-TK is a cell cycle-dependent suicide gene that needs to function based on ganciclovir, iC9 is recommended for CAR-T cell therapy rather than HSV-TK (Tiberghien et al., 2001). Existing clinical trials using iC9 CAR-T cells include NCT03016377, NCT03594162, NCT03696784, and NCT03579927 (Andrea et al., 2020).

## Summary and prospects

CAR-T cell therapy is one of the most attractive treatment options for patients with r/r hematological malignancy. It also has considerable potential for treating other malignancies. However, CRS and severe adverse reactions in the organ system after CAR-T cell infusion can be fatal to patients, and are also essential factors preventing the early application of CAR-T in hematological malignancies. It is especially urgent to clarify the symptoms, pathophysiology, grading criteria, and therapeutic measures of related adverse reactions to ensure the safety and efficacy of CAR-T immune cell therapy.

For severe CAR-T cell-related adverse reactions, rapid progression of toxicity causes irreversible harm to the body. Therefore, early identification and intervention effectively reduce the incidence and mortality of severe adverse reactions. In addition to considering preventive and therapeutic measures for CRS-related severe adverse reactions after CAR-T infusion, the design of the structure of CAR-T (such as multiple target antigens, adding “suicide” genes), manufacturing methods of CAR-T cell products, and optimizing the composition and infusion dose of CAR-T cell products curbs the incidence of severe adverse reactions concurrent with CRS from the cause. The target antigen of CAR-T therapy is key to treating cancer and is the main reason for target or off-target effects. Designing dual-target antigens are beneficial for reducing toxicities caused by insufficient target expression specificity. Multiple CAR-T cell clinical trials (NCT02903810, NCT03098355, NCT03241940) targeting CD19 and CD22 are currently ongoing (Annesley et al., 2018). With the development of relevant research and the advancement of gene screening technology, understanding the specific mutations in tumors and selecting tumor-specific antigens for precise, personalized treatment will substantially benefit the patients. In addition, using CRISPR/Cas9 technology to control the production of pro-inflammatory mediators in CAR-T cells is another effective way to improve severe CRS-related adverse reactions (Salas-Mckee et al., 2019). However, these methods are still in the research phase and need to be tested in clinical trials, which is a lengthy and costly process.

Unlike surgery, chemoradiotherapy, and immune checkpoint inhibitors, T-cells in CAR-T therapy can exist in the body for up to 10 years (Scholler et al., 2012). However, CAR-T therapy first approved in the market only 5 years; therefore, understanding the mechanisms of related adverse reactions and effectively treating them is currently ongoing. Unresolved issues in CAR-T therapy include the following:

• Siltuximab, etanercept, infliximab, fontolizumab, anakinra, itacitinib, ruxolitinib, dasatinib, and ibrutinib may be effective in the treatment of CRS; however, clinical trials on these are sparse.
• Although the ZUMA-1 cohort 6 study showed that prophylactic use of corticosteroids in patients receiving axicabtagene ciloleucel for LBCL could reduce the incidence of grade ≥3 CRS and ICANS, there is no clear consensus on the requirement of the prophylactic use of corticosteroids for all patients receiving CAR-T cell infusion (Oluwole et al., 2021).
• Some progress has been made in research on biomarkers for predicting the toxicity of CAR-T therapy, but there are no precise biomarkers to predict its efficacy. The main difficulty is that different CAR-T products, patient ages, and measurement times after infusion may require distinct cell biomarkers.
• Whether the long-term high-dose use of corticosteroids affects the effect of CAR-T therapy requires further research.
In summary, close clinical monitoring and early prevention, diagnosis, and treatment of adverse reactions are vital to reducing adverse events. With the concerted efforts of researchers, doctors, pharmacists, and nurses, CAR-T therapy will eventually become a safer and more effective conventional treatment for cancers.
